# Diagnostic Performance of Complementary Serum and Cerebrospinal Fluid Multiplex PCR for Acute Bacterial Meningitis

**DOI:** 10.3390/diagnostics16172800

**Published:** 2026-08-31

**Authors:** Lema Ayele, Daniel Asrat, Aminu Seman, Jemal Aman, Muluwork Tefera, Derso Wale Mesele, Ashenafi Alemu, Andargachew Mulu, Dawit Hailu Alemayehu, Adane Mihret, Getachew Tesfaye Beyene

**Affiliations:** 1 Armauer Hansen Research Institute (AHRI), Addis Ababa P.O. Box 1005, Ethiopia; ashenafialemu07@gmail.com (A.A.); andargachew.mulu@ahri.gov.et (A.M.); dawit.hailu@ahri.gov.et (D.H.A.); adane.mihret@ahri.gov.et (A.M.); 2 Department of Medical Laboratory Science, College of Health Science, Arsi University, Asella P.O. Box 193, Ethiopia; 3 Department of Medical Microbiology, Immunology, and Parasitology, Addis Ababa University, Addis Ababa P.O. Box 1176, Ethiopia; asratdan@gmail.com (D.A.); aminu.seman@aau.edu.et (A.S.); 4 Department of Medical Laboratory Science, College of Health Science, Madda Walabu University, Robe P.O. Box 247, Ethiopia; jamaaluna@gmail.com; 5 Department of Pediatrics and Child Health, College of Health Science, Addis Ababa University, Addis Ababa P.O. Box 1176, Ethiopia; muluworktef@yahoo.com; 6 Department of Medical Laboratory Science, College of Medicine and Health Science, Wolkite University, Wolkite P.O. Box 07, Ethiopia; derso.wale@wku.edu.et

**Keywords:** bacterial meningitis, cerebrospinal fluid, serum, polymerase chain reaction, diagnosis, Ethiopia

## Abstract

**Background**: Developing molecular tools for the diagnosis of bacterial meningitis is crucial for improving the diagnosis and initiation of prompt treatment of patients. This study aimed to evaluate the diagnostic performance of cerebrospinal fluid and serum multiplex PCR compared with conventional diagnostic methods for detecting bacterial pathogens among patients clinically suspected of bacterial meningitis. **Methods**: A cross-sectional study was conducted at Tikur Anbessa Specialized Hospital and Yekatit 12 Hospital Medical College from August 2023 to February 2024. A total of 202 patients who were suspected of having meningitis were included in the study. Conventional laboratory analysis, including Gram staining and bacterial culture from CSF, was performed. CSF and serum PCR for the detection of *Neisseria meningitidis, Haemophilus influenzae*, *Streptococcus pneumoniae*, *Streptococcus agalactiae*, *Escherichia coli*, *Staphylococcus aureus*, *Listeria monocytogenes*, and *Klebsiella pneumoniae* were performed. The agreement between tests used to detect etiologic agents of bacterial meningitis was determined by *McNemar’s Test* and Cohen’s kappa statistic. **Results**: Of the 202 study participants, 109 (54.0%) were male, and 178 (88.1%) were pediatric, with the majority being infants (mean age: 1.45 ± 0.69 years). Poor feeding was the most common clinical presentation at 151/202 (74.8%), followed by high fever at 144/202 (71.3%), and loss of consciousness at 134/202 (66.3%). Among the 202 CSF samples, nine (4.5%) and 11 (5.4%) tested positive for culture and Gram stain, respectively. Bacterial DNA was detected in 54/202 (26.7%) of the CSF samples and 93/202 (46.0%) of the serum samples. *E. coli* was detected in 30/54 (55.5%) of CSF samples and 57/93 (61.2%) of serum samples, followed by *S. pneumoniae*, which was detected in 8/54 (14.8%) of CSF samples and 27/93 (29.0%) of serum samples. The overall agreement between CSF and serum PCR is 60.9% (123/202), with a positive percent agreement of 62.96% (34/54), and a negative percent agreement of 60.14% (89/148). McNemar’s test indicated a significant difference between the two tests (*p* < 0.0001), and Cohen’s kappa statistic showed slight agreement (κ = 0.188, 95% CI: 0.109–0.293). **Conclusions**: Bacterial DNA was detected more often in serum multiplex PCR than CSF PCR or culture, suggesting its potential role as a complementary diagnostic tool for epidemiological surveillance, particularly in resource-limited settings where lumbar puncture may not always be feasible. However, serum PCR should not replace CSF analysis for definitive diagnosis; rather, it can serve as a screening or surveillance tool, with positive results requiring clinical correlation and, where possible, CSF confirmation. A relatively small sample size, reliance on conventional PCR, absence of comparison with quantitative PCR, potential blood contamination, lack of sequencing, and lack of long-term outcome data limit the generalizability of our findings. Future investigations should include a large sample size in conjunction with confirmatory culture techniques to enhance the robustness and validity of the findings.

## 1. Introduction

Bacterial meningitis (BM) continues to be a significant infectious disease globally, despite the progress made in treatment and vaccination [1]. Regularly, meningitis is suspected on the basis of the patient’s clinical presentation. However, decisions that rely on clinical presentation are often problematic and prone to incorrect diagnoses, leading to incorrect treatment. Thus, rapid and accurate diagnosis is required for the proper management of patients [2] and for timely antibiotic prophylaxis for contacts [3].

According to national and regional surveillance data, *Neisseria meningitidis* (*N. meningitidis*), *Streptococcus pneumoniae* (*S. pneumoniae*), and *Haemophilus influenzae* type b (Hib) are the most prevalent pathogens in Ethiopia’s bacterial meningitis epidemiology [4,5]. *Escherichia coli* (*E. coli*), however, has become a major cause in recent years, particularly in neonates [6,7].

Diagnosing BM is very challenging, especially in low-resource settings such as Ethiopia, due to late patient presentation [8], limited diagnostic facilities [9], and the high level of technical skills required to collect CSF samples. Conventional tests, such as analyses of glucose and proteins in CSF and cell counts, are inexpensive and fast but not very specific [10,11]. However, a definitive diagnosis of BM can be made by culture, which requires several days and has low sensitivity compared with molecular methods [12]. Furthermore, the lumbar puncture procedure requires high levels of technical skill and is very invasive and painful to the patient.

The high rates of morbidity and mortality linked to meningitis, particularly in children, are partly caused by delayed detection and diagnosis. Thus, developing a fast and reliable diagnostic method for the detection of meningitis agents is clinically relevant. In this context, molecular methods offer several advantages over culture-based methods and analyses of other CSF parameters, including increased sensitivity, specificity, speed, and efficiency [13,14]. Reports show variable results of the use of blood PCR, for example, as early as 1996, a study demonstrated promising findings of a PCR using a blood buffy coat and serum, with sensitivity ranging from 83 to 100% and specificity ranging from 87 to 100% [15], whereas another study reported low performance of serum PCR compared with CSF PCR [3]; both studies used a different target gene and focused on the detection of a single pathogen, *N. meningitidis*. Here, we compared the diagnostic performance of CSF and serum PCR for detecting meningitis agents. While some previous studies have used multiplex PCR for CSF or blood samples, our study uniquely compares the performance of both CSF and serum multiplex PCR in parallel for a broad range of bacterial pathogens, specifically within the Ethiopian context. Therefore, this study aimed to evaluate the diagnostic performance of CSF and serum multiplex PCR compared with conventional diagnostic methods for detecting bacterial pathogens among patients clinically suspected of bacterial meningitis.

## 2. Materials and Methods

### 2.1. Study Design and Period

An institution-based cross-sectional study was conducted at Tikur Anbessa Specialized Hospital (TASH) and Yekatit 12 Hospital Medical College (Y12HMC) from August 2023 to February 2024.

### 2.2. Study Area and Setting

The study was carried out at the diagnostic microbiology laboratories of two major institutions in Ethiopia: Tikur Anbessa Specialized Hospital and Yekatit 12 Hospital Medical College. Tikur Anbessa Specialized Hospital, which was established in 1972, is considered Ethiopia’s largest specialized hospital. It has around 800 beds and serves a large patient population of 370,000 to 400,000 per year. It has served as a teaching hospital of the School of Medicine under Addis Ababa University’s College of Health Sciences and provides training in a variety of medical fields. Yekatit 12 Hospital Medical College, under the supervision of the Addis Ababa City Administration Health Bureau, offers routine healthcare services to the city’s population, is a referral institution for other regions’ patients, and acts as a catchment referral for nearby health centers.

### 2.3. Study Population

The study population included all patients who were clinically suspected of having bacterial meningitis and indicated for diagnosis at the participating laboratories during the study period. Patients who did not provide both samples or had insufficient sample volumes were excluded from the study.

### 2.4. Inclusion and Exclusion Criteria

Patients were included if they met the following criteria: (1) clinically suspected of having bacterial meningitis according to the 2018 WHO standard recommendations for surveillance of meningitis preparedness and response to epidemics in Africa; (2) ≥28 days of age; deliberate methodological decision due to large CSF and blood volumes often needed for the full diagnostic panel; (3) underwent lumbar puncture as part of routine clinical care; and (4) provided both CSF and blood samples with sufficient volumes for the study procedures. Patients were excluded for the following reasons: (1) absence of both CSF and serum samples; (2) low sample volume for PCR analysis; (3) confirmed alternative non-bacterial diagnosis at time of enrolment.

### 2.5. Sample Size and Sampling Technique

**Figure 1:**
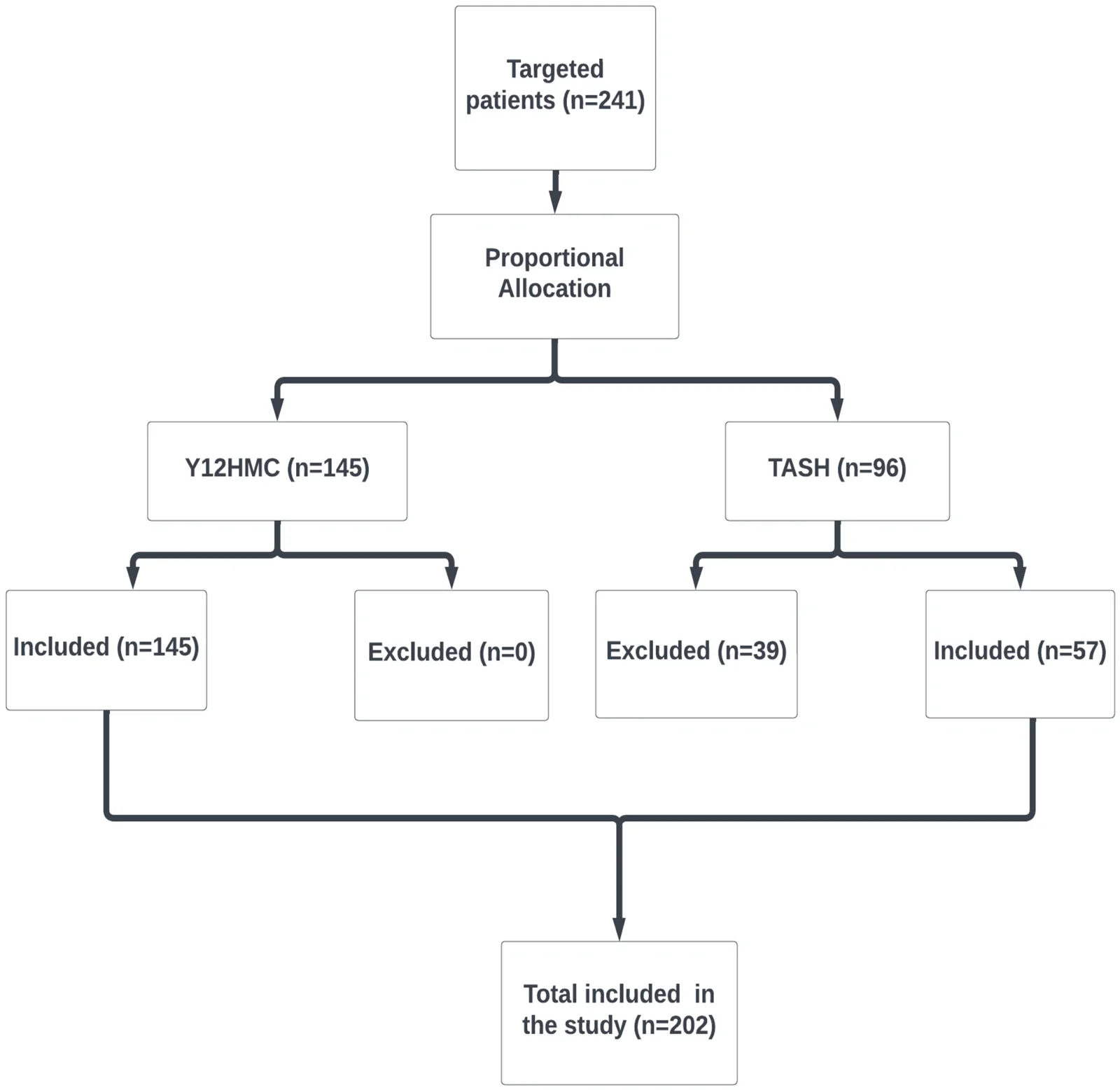
Patient recruitment flowchart (*N* = 202). A total of 241 suspected meningitis patients were initially enrolled from Tikur Anbessa Specialized Hospital (TASH) and Yekatit 12 Hospital Medical College (Y12HMC) between August 2023 and February 2024. Of these, 39 patients were excluded because they did not provide both CSF and serum samples or had insufficient sample volumes. The final analysis included 202 patients (145 from Y12HMC and 57 from TASH).

A nonprobability convenience sampling technique was employed. The total sample size was determined using a single-population proportion formula based on a 10% prevalence rate reported in a previous Ethiopian study [16]. A proportional allocation of samples was made by considering prior patient traffic at each study site in the past three months. In the prior three months, approximately 60 suspected meningitis patients visited Y12HMC, and approximately 40 patients visited TASH. By looking at this number, we allocated the total sample to each site accordingly: 145 patients to Y12HMC and 96 patients to TASH. A total of 145 suspected patients with meningitis from Y12HMC and 57 suspected patients were enrolled from TASH. Thirty-nine patients were excluded based on exclusion criteria at TASH (Figure 1).

### 2.6. Operational Definitions

The case definition for bacterial meningitis was based on the 2018 World Health Organization (WHO)-recommended standard for surveillance of meningitis preparedness and response to epidemics in Africa [17].

**Suspected meningitis** was considered when a patient presented with a sudden onset of fever (higher than 38.5 °C rectal or 38.0 °C axillary), neck stiffness, or other meningeal signs.

**Probable meningitis** was suspected for cases with a macroscopic aspect of cerebrospinal fluid that was turbid, cloudy, or purulent; with a CSF leukocyte count greater than 10 cells/µL; or positive Gram stain in CSF sample suggesting bacterial meningitis.

### 2.7. Demographic and Clinical Data Collection

A predefined questionnaire addressing demographic data and clinical investigation details defining bacterial meningitis was developed for this study by using the “National guideline on meningococcal meningitis surveillance and outbreak management” as a reference [18].

### 2.8. CSF Sample Collection

Two to three milliliters of CSF were aspirated into a sterile plastic tube by the attending physician or senior residents from the Department of Internal Medicine (for adult patients) and the Department of Pediatrics (for pediatric patients) following strict aseptic conditions. The collected samples were transported to the microbiology laboratories of TASH and Y12HMC at ambient temperature within ten minutes, and laboratory analysis was started within 30 min. A minimum of 1 mL of CSF sample for PCR was stored at −20 °C before conventional laboratory analysis started. The remaining was processed using conventional laboratory assays of CSF.

### 2.9. Blood Sample Collection

One to four mL blood samples were collected in serum separator tubes for patients undergoing lumbar puncture by nurses as soon as CSF was collected. The blood samples were placed at room temperature and allowed to clot. Samples were centrifuged at 6000 rpm for 10 min. The serum for PCR was transferred via a sterile Pasteur pipette to a cryotube and stored at −20 °C.

### 2.10. Conventional Laboratory Analyses of CSF

The samples were processed according to standard laboratory procedures for Gram staining, bacterial culture, and manual CSF white blood cell (WBC) counts. CSF was visually inspected for clarity, cloudiness, turbidity, and hemorrhage. All the samples were centrifuged at 3000 rpm for 15 min. The supernatant was discarded, and the sediment was resuspended by pipetting. Following Gram staining, a drop of suspension was inoculated on solid agar plate’s blood agar, chocolate agar, or MacConkey agar plates (Oxoid Ltd., Basingstoke, Hampshire, UK). The chocolate and blood agar plates were incubated in a candle jar with 5% CO_2_ concentration to create a microaerophilic condition, and MacConkey agar plates were incubated aerobically. All the agar plates were incubated at 37 °C for 24–48 h. Pure isolates were identified by their colony characteristics on their respective media, Gram staining, and confirmed by biochemical tests. Gram-negative rods were identified by indole production, hydrogen sulfide (H_2_S) production, citrate utilization, motility test, urease test, oxidase, and triple sugar iron (TSI) agar, while Gram-positive cocci were identified by catalase, coagulase, bacitracin, and optochin susceptibility tests. For the optochin susceptibility test, disks manufactured by Micro Master (Camarillo, CA, USA) were utilized in the presence of 5% CO_2_ to provide the best environment for the bacteria to grow [19]. American Type Culture Collection (ATCC) reference strains, *N. meningitidis* ATCC 13090; *S. pneumoniae* ATCC 49619; *H. influenzae* ATCC 10211; *S. agalactiae* ATCC 12386; *E. coli* ATCC 25922; *S. aureus* ATCC 25923; *L. monocytogenes* ATCC 19115; and *K. pneumoniae* ATCC 13883, were obtained from the Ethiopian Public Health Institute (EPHI), Addis Ababa, Ethiopia, and used to validate media and biochemical identification for the pathogens.

### 2.11. Nucleic Acid Extraction

DNA was extracted using a Bioer NPA-32P automated nucleic acid purification machine (Hangzhou Bioer Technology Co., Ltd., Hangzhou, China), as recommended by the manufacturer, utilizing a 96-well plate pre-loaded with Lysis and Wash Buffers. Briefly, the 96-well plate was kept at room temperature and inverted upside down three times, and centrifuged to avoid reagents adhering to the wall of the tubes. Then, for both sample types (CSF and serum), a fixed volume of 300 µL of the CSF and serum samples were transferred to a 96-deep well plate, followed by placing the 96-deep well plate into the instrument. The positive control for all targeted bacteria was extracted from the corresponding ATCC strains via the same procedure applied to the samples. Nuclease-free water was extracted as a negative control along with the samples. The extracted nucleic acid was eluted into a volume of 50 µL, and its concentration and purity were measured using 1 µL of the eluate by a NanoDrop spectrophotometer (Thermo Fisher Scientific, Waltham, MA, USA) at absorbance of 260 nm and A260/280 ratios. The extracted DNA showed good purity for subsequent PCR applications, with A260/280 ratios ranging from 1.8 to 2.0. The extracted nucleic acid was stored at −20 °C until use.

### 2.12. Multiplex PCR Assay Development

The assay optimization started with a uniplex PCR for the detection of individual bacterial etiologies of meningitis using ATCC strains of the target bacteria: *E. coli*, *S. aureus*, *K. pneumoniae*, *N. meningitidis*, *S. pneumoniae*, *H. influenzae*, *L. monocytogenes*, and *S. agalactiae*. Similarly, the optimization of the multiplex PCR procedure was performed with ATCC strains. In order to perform the final diagnostic testing of clinical samples, a multiplex PCR approach was used. Based on the primers’ properties, the bacteria were divided into two multiplex panels: Group 1: *N. meningitidis*, *S. pneumoniae*, *H. influenzae*, and *S. agalactiae*; Group 2: *E. coli*, *S. aureus*, *L. monocytogenes*, and *K. pneumoniae*. Following this, two multiplex PCR master mixes, each containing specific primers at the concentration indicated in Table 1, were prepared. The annealing temperature were optimized to obtain the maximum sensitivity and specificity by using gradient PCR from 53 °C to 63 °C.

Finally, the multiplex PCR assay was performed using Platinum Taq polymerase (Invitrogen, Waltham, MA, USA) and was amplified in a thermocycler (T100 LASEC; BIO-RAD, Hercules, CA, USA). Different bacteria were detected by targeting specific genes of the target organisms (Table 1). As indicated above, the bacteria were grouped into two multiplex panels. For both multiplex PCR groups, each reaction was prepared in a final volume of 25 μL, consisting of 2.5 μL of 10× PCR buffer, 1.2 μL MgCl_2_, 1 μL dNTPs, 1 μL of each specific primer (1 μL forward and 1 μL reverse), 0.2 μL Platinum Taq polymerase, 5 μL DNA templates, and nuclease-free water. The final primer concentrations in each multiplex reaction were 0.4 μM for each primer. The cycling conditions were the same for both tests: initial denaturation at 94 °C for 3 min, followed by 35 cycles consisting of final denaturation at 94 °C for 10 s, an annealing step at 60 °C for 30 s, an extension step at 72 °C for 30 s, a final extension at 72 °C for 5 min, and an infinite hold at 12 °C. All the PCR products were visualized via a 2.5% agarose gel with ethidium bromide under UV light using CS-DARKROOM, Cleaver Scientific, Rugby, UK.

### 2.13. Data Quality Assurance

Data collectors were trained on the data collection tools, including the questionnaire. Unique identifiers, including date, time, location, and collector name, were assigned to the samples/patients, and the data were accurately recorded. The samples were transported to TASH and Y12HMC microbiology laboratories as soon as possible and processed following established laboratory protocol and guidelines, which include Gram staining and culture. Quality controls for inoculation, incubation, and reagents were maintained. The PCR samples were subsequently transported to AHRI and kept at −80 °C until they were used for PCR analysis. Quality control checks were performed for the extraction. Extraction controls (ATCC and nuclease-free water) were used to monitor extraction efficiency. A standardized PCR protocol and a master mix were used. Positive (verifies that the PCR reagents, including primers and enzymes, function correctly) and negative (for checking contamination in the PCR setup) controls for each PCR run were implemented. Each laboratory procedure (DNA extraction, master mix preparation, template addition, and sample storage) was carried out in a separate room to avoid cross-contamination.

### 2.14. Statistical Analysis

The data were entered into Epi Info v7.2.5 (CDC, Atlanta, GA, USA), then transformed and analyzed using IBM SPSS Statistics, Version 26.0 (IBM Corp., Armonk, NY, USA) statistical software. The results of the analysis were presented in figures or tables where appropriate. Frequency and percentage were used to summarize categorical variables. *McNemar’s* test compared paired proportions between CSF and serum PCR results. Cohen’s kappa was calculated for inter-reader agreement, which was classified as follows: ≤0.00, no agreement; 0.00–0.20, none to slight; 0.21–0.40, fair; 0.41–0.60, moderate; 0.61–0.80, substantial; >0.81, almost perfect [24]. A two-proportion Z-test using MedCalc software version 23.4.5 (MedCalc Software Ltd, Ostend, Belgium) [25]. Statistical significance was set at *p* < 0.05.

## 3. Results

### 3.1. Sociodemographic Characteristics

Two hundred and two patients suspected of having meningitis were included in the study; 109 (54.0%) were male. The majority, 178/202 (88.1%), were pediatric: 134 (66.3%) were infants (1–12 months), and 44 (21.8%) were children (1–15 years). There were 24 (11.9%) adults (≥16 years) (Table 2). The mean age was 1.45 ± 0.69 years, indicating that the study population was predominantly infants.

### 3.2. Clinical Presentation of the Study Participants

The most common clinical presentation shown by the study participants was poor feeding at 151/202 (74.8%), followed by high fever at 144/202 (71.3%) and loss of consciousness at 134/202 (66.3%) (Table 3).

### 3.3. CSF WBC Count, Gram Stain, and Culture

Among the 202 CSF samples, WBC count >10 cells/mm^3^ was observed in 52 (25.7%) samples. Gram staining revealed bacteria in 11 (5.4%) samples, and bacterial culture was positive in only nine (4.5%) samples. A history of antibiotic use before admission was reported by 140 (69.3%) patients, and 59 (29.2%) had a history of vaccination (Table 4).

### 3.4. Assay Optimization

**Figure 2:**
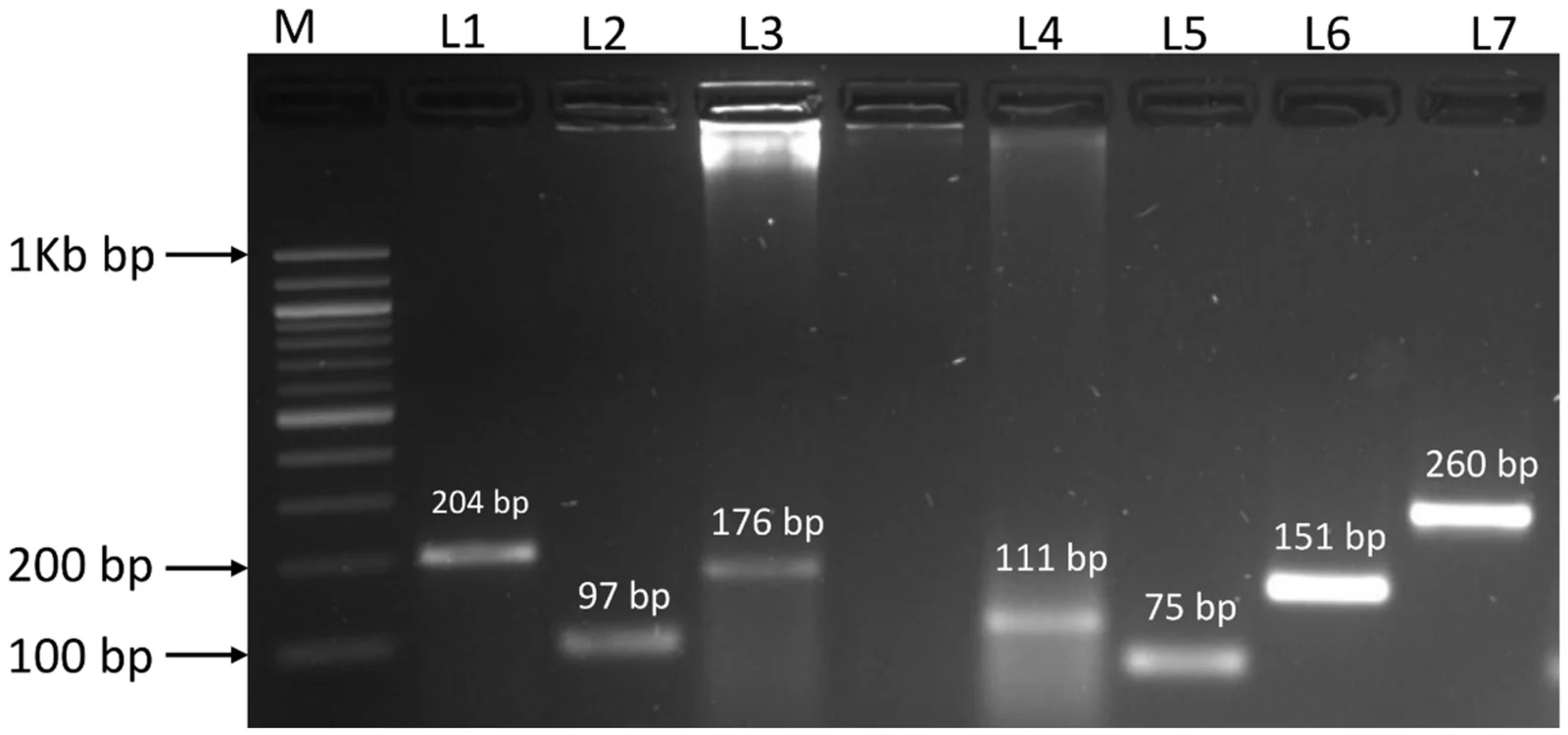
Representative gel electrophoresis image of uniplex PCR optimization. The gel image presents the PCR product amplification corresponding to: M—100 bp DNA ladder, L1—*E. coli*, L2—*S. aureus*, L3—*K. pneumoniae*, L4—*N. meningitidis*, L5—*S. pneumoniae*, L6—*H. influenzae*, and L7—*S. agalactiae*.

**Figure 3:**
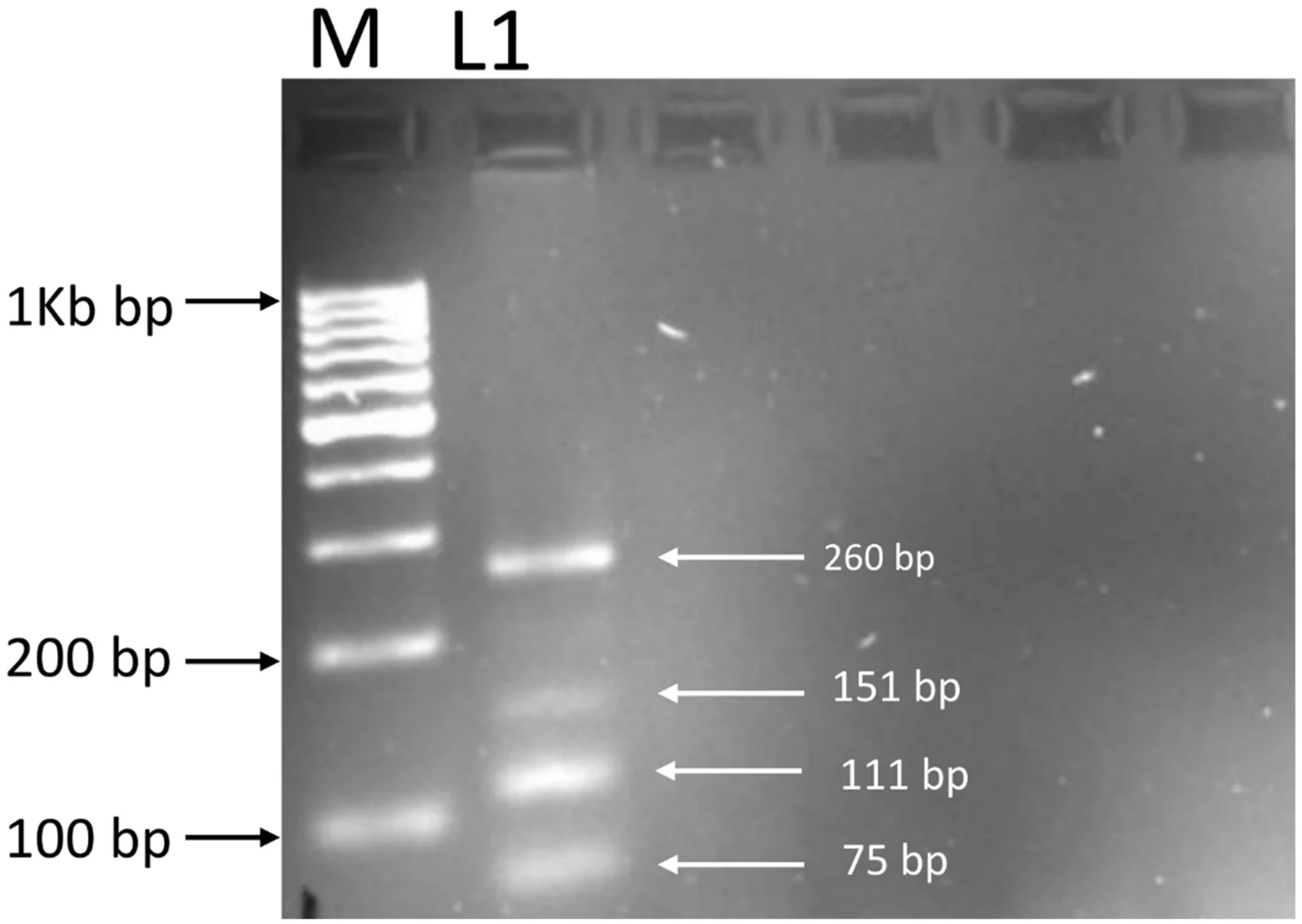
Representative gel electrophoresis of multiplex PCR assay for Group 1 pathogens. The gel image demonstrates: M—100 bp DNA ladder, and L1—the PCR product amplification corresponding to 111 bp *N. meningitidis*, 75 bp *Streptococcus pneumoniae*, 151 bp *H. influenzae*, and 260 bp *S. agalactiae*.

**Figure 4:**
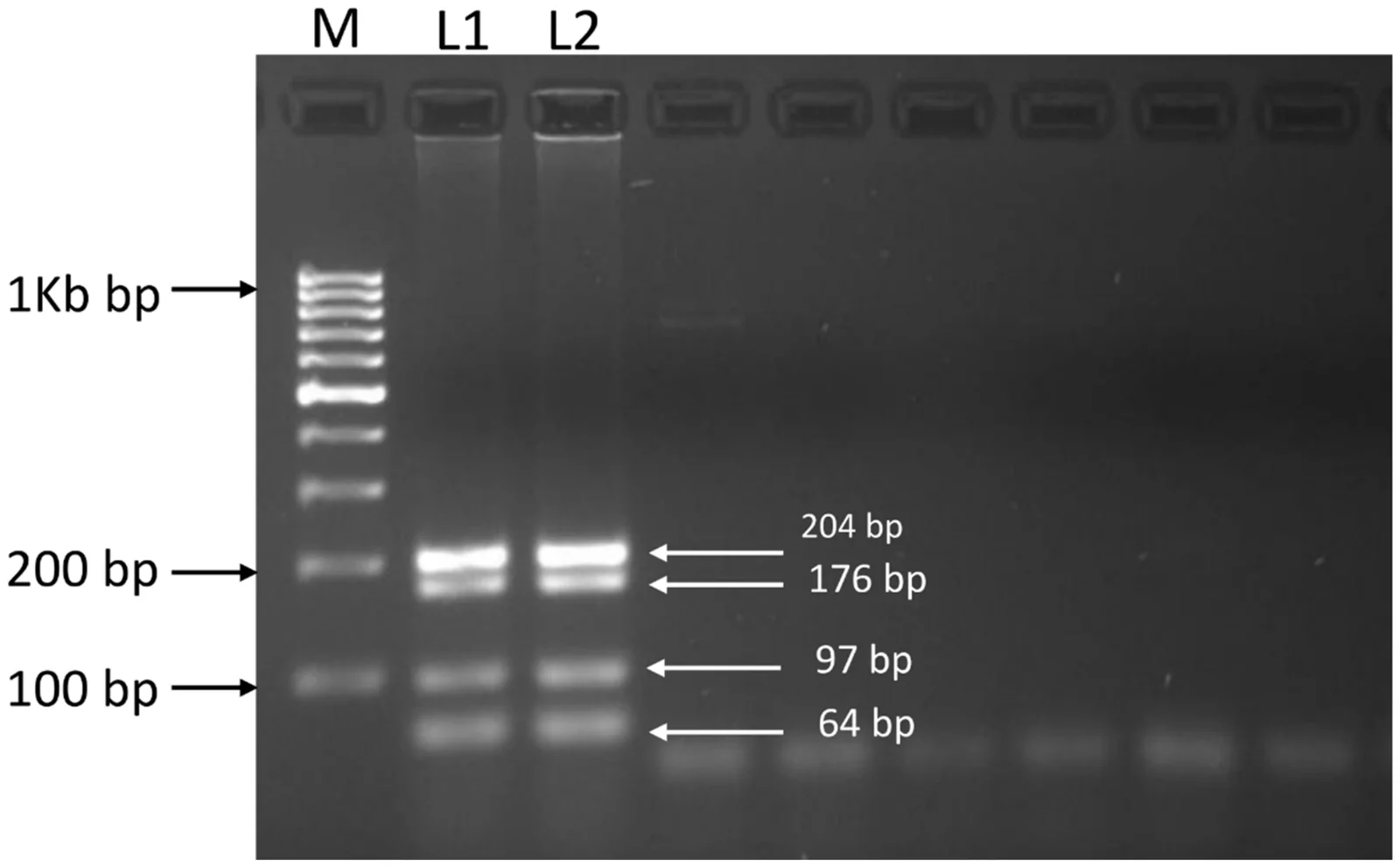
Representative gel electrophoresis of multiplex PCR assay for Group 2 pathogens. The gel images in both L1 and L2 show PCR product amplifications corresponding to: M—100 bp DNA ladder; 64 bp *L. monocytogenes*; 97 bp *S. aureus*; 176 bp *K. pneumoniae*; and 204 bp *E. coli*.

Protocol optimization for uniplex PCR was assured as all positive controls for the eight targeted bacterial pathogens were successfully amplified and visualized as a single band (Figure 2). The optimization for multiplex PCR was assured as all the positive controls were successfully amplified, and the four bands were visualized in a single reaction for both groups of bacteria. There were no amplified bands for the negative controls. PCR products for Group 1 and Group 2 are presented in Figure 3 and Figure 4, respectively.

### 3.5. Pathogens Detected Using CSF PCR

**Figure 5:**
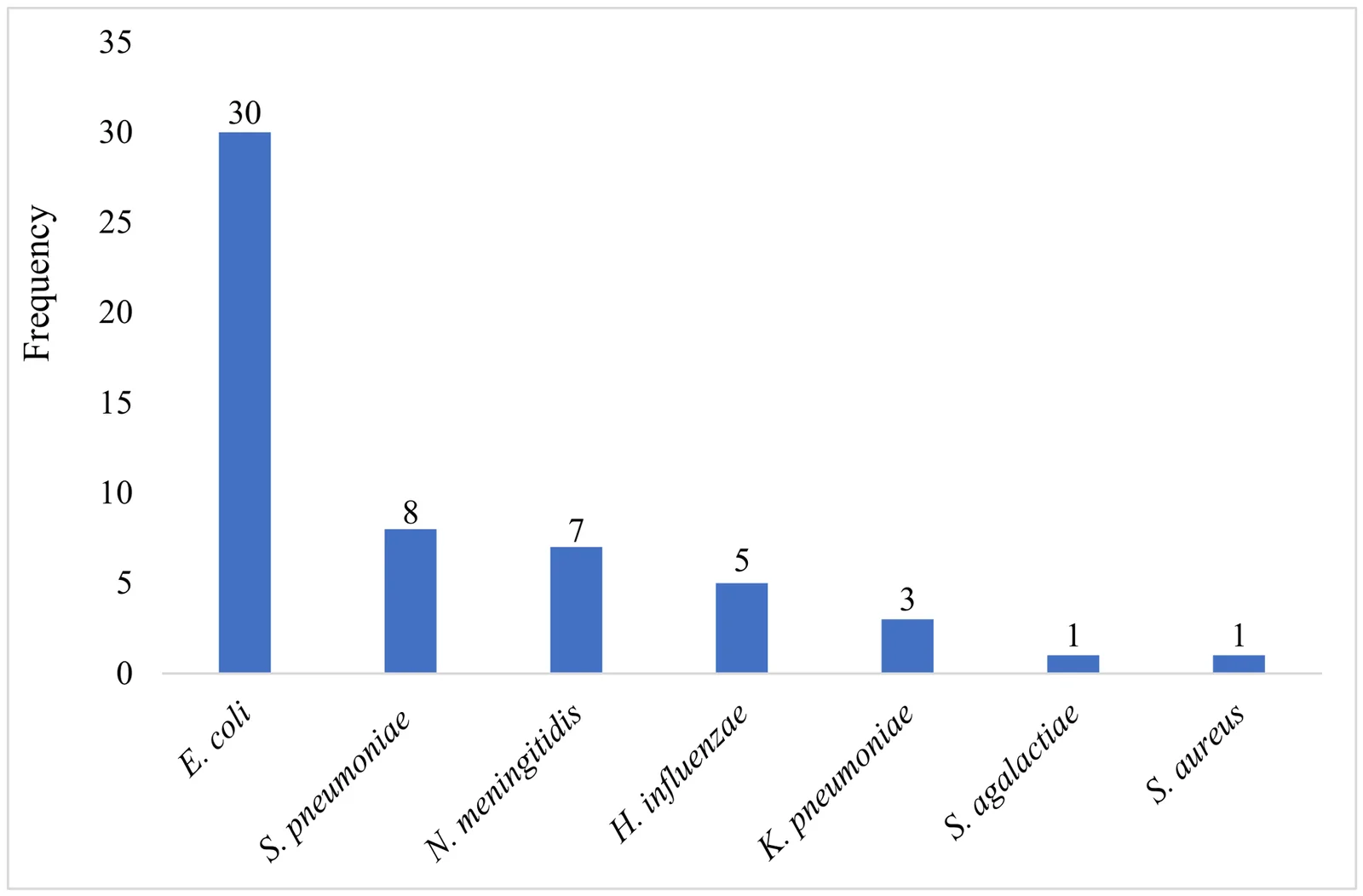
Frequency of pathogenic bacterial species detected using CSF PCR (*n* = 54 positive samples). Among 202 CSF samples analyzed, 54 (26.7%) were positive by multiplex PCR. *E. coli* was the most common pathogen detected (30/54; 55.5%), followed by *S. pneumoniae* (8/54; 14.8%), *N. meningitidis* (7/54; 13.0%), *H. influenzae* (5/54; 9.3%), and *K. pneumoniae* (3/54; 5.6%). Single coinfections (*E. coli* with *N. meningitidis*) were detected in one CSF sample. Total pathogen detections exceed the number of positive samples due to coinfection.

Two hundred and two CSF samples were analyzed by conventional multiplex PCR, and DNA was amplified from 54 (26%) samples. *E. coli* was the most common pathogen detected in 30/54 (55.5%) samples, followed by *S. pneumoniae,* which was detected in 8/54 (14.8%) of the PCR-positive samples. Multiplex PCR identified double infections in 1 CSF sample (*E. coli* with *N. meningitidis*). Overall, a total of 55 bacterial pathogens were detected in 54 CSF-positive samples (Figure 5).

### 3.6. Pathogens Detected Using Serum PCR

**Figure 6:**
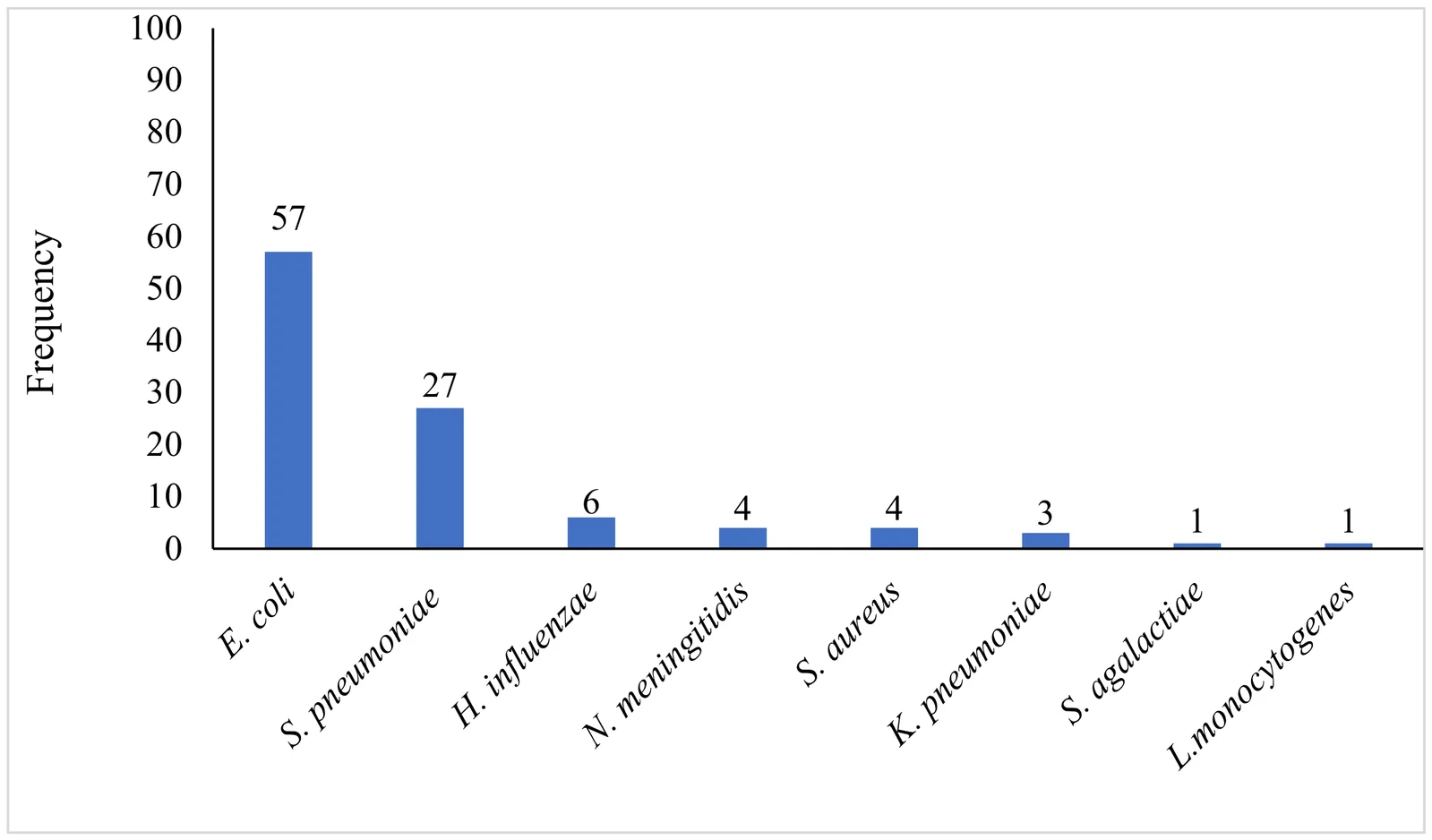
Frequency of pathogenic bacterial species detected using serum PCR (*n* = 93 positive samples). Among 202 serum samples analyzed, 93 (46.0%) were positive by multiplex PCR. *E. coli* was the most common pathogen detected (57/93; 61.2%), followed by *S. pneumoniae* (27/93; 29.0%), *H. influenzae* (6/93; 6.5%), *N. meningitidis* (4/93; 4.3%), *S. aureus* (4/93; 4.3%), and *K. pneumoniae* (3/93; 3.2%). Coinfections were detected in 10 serum samples (details provided in text). Total pathogen detections exceed the number of positive samples due to coinfections.

Two hundred and two serum samples were analyzed via conventional multiplex PCR, and DNA was amplified from 93 (46%) samples. *E. coli* was the most common pathogen detected in 57/93 (61.2%) of the serum-positive samples, followed by *S. pneumoniae,* which was detected in 27/93 (29%) samples. Multiplex PCR revealed double infections in 10 serum samples, including *E. coli* with *S. pneumoniae* (3 cases), with *K. pneumoniae* (1 case), with *H. influenzae* (1 case), with *N. meningitidis* (1 case), with S. aureus (1 case), and *S. pneumoniae* with *S. aureus* (2 cases) and with *K. pneumoniae* (1 case). Overall, a total of 103 bacterial pathogens were detected in 93 serum-positive samples (Figure 6).

### 3.7. Comparison of Serum-Based PCR, CSF PCR, and Conventional Methods

**Figure 7:**
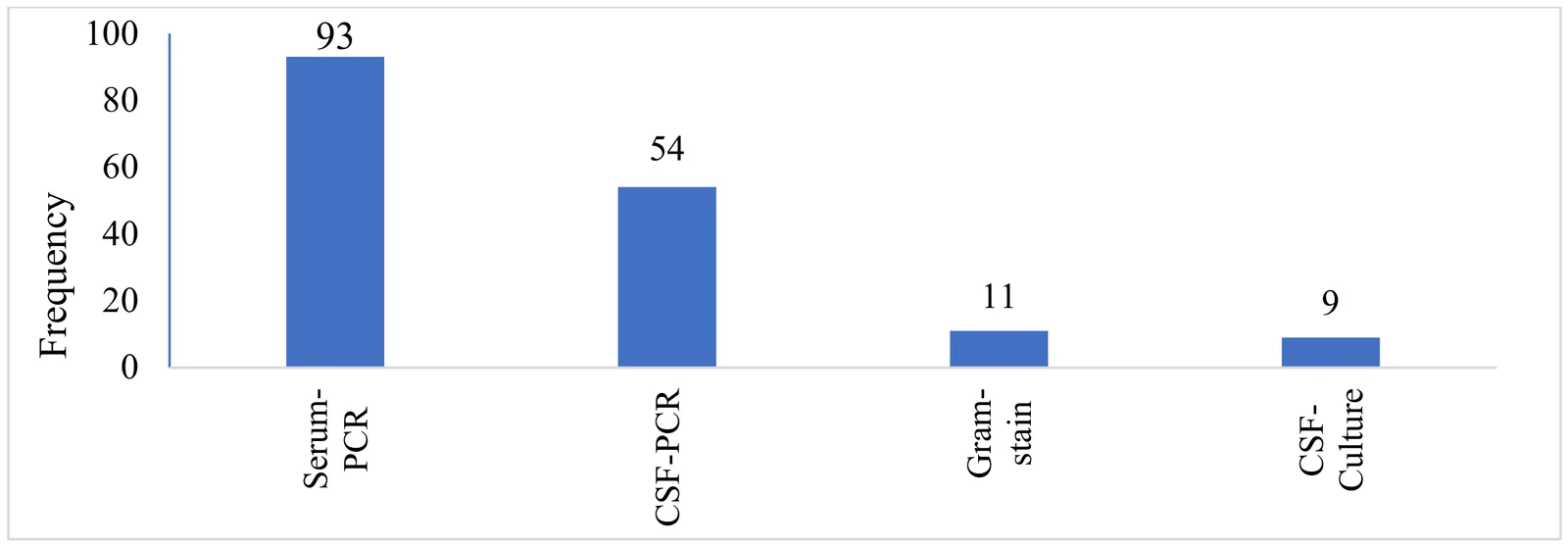
Comparison of positivity rates between diagnostic methods. Bar chart comparing the positivity frequency of serum PCR (46.0%; 93/202), CSF PCR (26.7%; 54/202), Gram stain (5.4%; 11/202), and bacterial culture (4.5%; 9/202). PCR-based methods detected bacterial DNA at higher rates than conventional methods.

Among the 202 CSF samples, 9 (4.5%) and 11 (5.4%) tested positive for culture and Gram stain, respectively. Bacterial DNA was detected in 54/202 (26.7%) of the CSF samples and 93/202 (46.0%) of the serum samples (Figure 7).

### 3.8. Comparison of Pathogens Detected via CSF and Serum PCR

A total of 30 *E. coli* were detected in CSF samples, whereas 57 were detected in serum samples, with 17 concordant positive cases. Eight *S. pneumoniae* cases were detected in CSF, while 27 were detected in serum, with five concordant positives (Table 5).

### 3.9. Agreement Between CSF and Serum Samples Using Multiplex PCR

The overall agreement between CSF and serum PCR was 60.9% (34 + 89)/202 × 100. The positive percent agreement was 62.96% (34/54 × 100), and the negative percent agreement was 60.14% (89/148 × 100). McNemar’s test indicated a significant difference between the two tests (*p* < 0.0001). Cohen’s kappa statistic showed slight agreement (κ = 0.188, 95% CI: 0.109–0.293) (Table 6).

### 3.10. Site-Specific Positivity Rates

The CSF PCR positivity rate was 26.73% (54/202), whereas the serum PCR positivity rate was 46.0% (93/202). Accordingly, the total difference in positivity between the two sample types was 19.31%, with a 95% confidence interval ranging from 10% to 28%. Statistical test analysis yielded a Chi-squared value of 16.230, corresponding to a highly significant *p*-value of 0.0001.

### 3.11. Antibiotic Treatment Before Sample Collection and PCR Findings

Among the 140 patients who had taken antibiotics before sample collection, 99 were PCR-positive in either CSF or serum. Of these, 39 were CSF PCR-positive: *E. coli* 20/39 (51.2%), *N. meningitidis* 6/39 (15.4%), *S. pneumoniae* 8/39 (20.5%), *H. influenzae* 3/39 (7.7%), and *K*. *pneumoniae* 2/39 (5.1%). Sixty serum samples were PCR-positive: *E. coli* 39/60 (65.0%), *S. pneumoniae* 16/60 (26.7%), *H. influenzae* 6/60 (10.0%), *N. meningitidis* 3/60 (5.0%), *S. aureus* 3/60 (5.0%), and *K. pneumoniae* 1/60 (1.7%) (Table 7).

## 4. Discussion

This study compared the performance of serum-based PCR and CSF PCR and conventional methods, including Gram stain and bacterial culture, and revealed that serum-based PCR was more sensitive than CSF PCR and routine bacterial culture in our setting, and also showed that the performance of all PCRs was generally superior to that of routine bacterial culture. Furthermore, serum-based PCR is more convenient than CSF PCR and routine bacterial culture, reducing the turnaround time, pain, and cost. Overall, via CSF-PCR, we identified the common bacterial causative agents of meningitis, and the yield was 54/202 (26%). This finding is in agreement with similar studies conducted elsewhere [16,26,27,28,29]. Our findings reveal that PCR-based detection outperformed conventional diagnostic methods by 26%, and the classical culture and Gram staining demonstrate alarmingly low sensitivity. This implies that a heavy reliance on these methods results in a substantial underestimation of the true bacterial meningitis burden in Ethiopia. This necessitates a shift in national surveillance protocols toward molecular confirmation to address the high rate of false-negative reports linked with the conventional methods.

Notably, the PCR positivity rates in our study (26.7% in CSF and 46.0% in serum) were much higher than the 10% prevalence rate used for sample size calculation, which was based on earlier Ethiopian studies predominantly using conventional culture methods [16]. This disparity highlights the increased sensitivity of molecular techniques compared to culture-based methods, particularly in settings where a substantial proportion of patients (69.3% in our cohort) had received antibiotics before sample collection. Despite the higher-than-expected positivity rate, we performed a post hoc power analysis to confirm that our study had sufficient power for the primary comparisons. The power to find the observed difference in CSF and serum PCR positivity rates (difference = 19.31%) with a sample size of 202 was >99% (α = 0.05, two-tailed) using McNemar’s test for paired proportions. Similarly, the power was >99% for comparing PCR positivity rates with culture (difference = 22.2% for CSF PCR vs. culture and 41.5% for serum PCR vs. culture). These results support the sample size being sufficient for the primary comparisons in this study. However, the discrepancy between the estimated and observed prevalence also highlights the need for updated epidemiological data in the Ethiopian context to guide future study designs and surveillance strategies.

Statistical analysis using McNemar’s test (*p* < 0.0001) demonstrated that paired binary data (CSF PCR versus serum PCR from the same patient) are significantly different between the two sample types. The uneven distribution of discordant pairs (59 serum-positive/CSF-negative vs. 20 CSF-positive/serum-negative) indicates that serum PCR is more likely to find bacterial DNA than CSF PCR in this population. Furthermore, Cohen’s kappa statistic (kappa = 0.188, 95% CI: 0.109–0.293) revealed slight agreement between the two tests according to Landis and Koch criteria (0.01–0.20 = slight agreement). The predominance of serum-positive/CSF-negative cases highlights the potential of serum PCR as an initial screening tool rather than a standalone diagnostic substitute for CSF investigation. Because serum PCR detects bacterial DNA in the bloodstream, it cannot anatomically distinguish concurrent bacteremia from isolated meningeal infection. Therefore, positive serum PCR results must be carefully correlated with clinical presentation and, where feasible, confirmed by CSF analysis to establish a definitive diagnosis of bacterial meningitis.

To the authors’ knowledge, this is the first study that used and compared serum PCR with CSF multiplex PCR targeting a wide range of bacteria causing meningitis. This study revealed that 46% of the serum samples tested positive for bacterial DNA, whereas only 26% of the CSF samples tested positive, with *Escherichia coli* (*n* = 30) being the predominant etiologic agent identified from CSF. This is similar to the findings from a local study that recently reported a high rate of *E. coli* [6], and studies from Nigeria and Kenya [30,31], which may be related to the high proportion of the pediatric age group, hospital exposure, antibiotic resistance, and the absence of vaccines against *E. coli*. 

The predominance of *E. coli* in our study, especially in infants (66.3%) and children (88.1%), has important clinical implications. The most common clinical presentations among the study participants were poor feeding (74.8%), high fever (71.3%), and loss of consciousness (66.3%). A high rate of these symptoms were seen in patients positive for *E. coli*. The higher number of *E. coli* positive serum (57 patients) than CSF (30 patients) samples probably indicates the presence of bacteremia prior to meningeal infection and supports the potential use of serum PCR as an early screening test in situations where lumbar puncture is difficult. Most importantly, the emergence of *E. coli* as the most common pathogen has important implications for empiric antibiotic selection, necessitating broader Gram-negative coverage in neonates, especially in the absence of a vaccine against *E. coli* in this vulnerable population. Also, the low culture recovery of *E. coli* may be due to the high rate of antibiotic use (69.3%) before the study, which further supports the utility of molecular methods for the detection of this pathogen.

We acknowledge that false-positive CSF PCR results may be caused by blood contamination during lumbar puncture. However, the higher CSF WBC counts seen in a minority of patients and the clinical presentation of patients (such as fever, poor feeding, and loss of consciousness) support the assumption that many CSF detections are actually meningeal infections. Although it does not prove meningeal invasion, the presence of bacterial DNA in serum samples is expected in bacteremic conditions.

In addition, seven bacterial species were identified via CSF-PCR; however, one additional bacterium, *L. monocytogenes*, was identified via serum PCR. The findings show that bacterial DNA is detected 20% more frequently via serum PCR than via CSF PCR in suspected cases of meningitis. Despite the limited use of serum-based PCR, one study reported similar sensitivity and specificity of detecting meningococcal DNA in buffy coat, which was comparable to that reported in confirmed meningococcal cases identified through culture or CSF analysis [15].

Similarly, the bacteria most frequently detected via serum PCR were *E. coli* (in 57 patients), followed by *S. pneumoniae* (27 patients), again highlighting the significance of *E. coli* and *S. pneumoniae* as leading causes of bacterial meningitis in the region. A significant difference was observed in the number of bacteria detected in the serum and CSF samples, whereas *E. coli* was detected in 57 of the serum samples, but only 30 were identified in the CSF samples. Similarly, *S. pneumoniae* was identified in 27 serum samples, whereas only eight were detected in CSF. These findings suggest the potential for the clinical utility of serum PCR in managing patients suspected of having meningitis. This is a promising step forward in the use of serum, which is a less invasive diagnostic procedure for patients.

Overall, studies indicate that Gram-negative rod bacteria are emerging as the predominant etiologic agents of meningitis, particularly in neonates [6,32]. Our findings show that *E. coli*, a key Gram-negative rod, is present in high proportions in both serum and CSF. Interestingly, the Gram-positive coccus, *S. pneumoniae*, was detected at higher frequencies in serum than in CSF. These results suggest that in Ethiopia and other resource-limited settings where Gram-negative rods predominate, the epidemiology of meningitis and CSF collection is technically and logistically challenging; serum should be utilized as a vital diagnostic complement to CSF.

Diagnosing bacterial meningitis is difficult, especially in the early stages of the disease, in infants and elderly individuals, or when the predominant signs and symptoms do not indicate meningeal infection. Clinical presentations, lethargy, impaired consciousness, convulsions, or a stiff neck have optimal performance in predicting BM in some settings, with 98% sensitivity and 72% specificity [33]. The major clinical presentations are consistent with those reported in other studies, where fever and loss of consciousness were common predictors of BM [34,35]. However, a wider range of clinical presentations should be taken into consideration when diagnosing BM and should be supported by laboratory tests, including molecular methods, for a definitive diagnosis of the infectious agent. In Ethiopia, similar to other developing countries, laboratory diagnosis of BM depends on classic methods, microscopy, latex agglutination, and isolation of pathogens from CSF or blood via culture. The latter requires 24–72 h and has limited diagnostic sensitivity, especially in patients with prior antibiotic treatment. Hence, developing rapid methods for diagnosing BM is crucial. Gram staining is a point-of-care diagnostic test, but it has low sensitivity and specificity [35]. In our study, only 11 (5.4%) of the 202 samples were positive for Gram stain, suggesting that Gram stain does not effectively capture all the cases of infection. Similarly, a very low culture recovery rate of pathogens was observed in our study (9; 4.5%), which is comparable to that reported in previous studies conducted in Ethiopia, with average recovery rates ranging from 5.2% to 6.9% [16,36,37,38,39]. However, our findings are lower than those of the studies performed elsewhere, ranging from 10% to 53% [40,41,42,43,44,45], which is potentially associated with prior antibiotic intake, as 69.3% of the patients were on different antibiotics before lumbar puncture.

## 5. Conclusions

This study presents a systematic comparison of CSF- and serum-based multiplex PCR for detecting bacterial etiologies of meningitis. While CSF remains the gold standard due to its anatomical specificity, serum PCR detected bacterial DNA more than CSF PCR or conventional culture. This suggests serum PCR’s potential as a complementary, less invasive tool for epidemiological surveillance, especially in resource-limited settings like Ethiopia, where lumbar punctures may not always be feasible. Serum PCR should not be used in place of CSF analysis for definitive diagnosis, but it can be used as a screening or surveillance tool; any positive results need to be correlated with the clinical picture and confirmed with CSF where possible.

## 6. Limitations

The major limitations of this study are that the majority of patients received different antibiotics before sample collection, with the exclusion of neonates (<28 days) being a deliberate methodological decision due to the small CSF and blood volumes, which are often insufficient for the full diagnostic panel; this limits generalizability to neonates, a small sample size, reliance on a conventional PCR technique, and a lack of comparison with quantitative PCR. Additionally, we acknowledge potential blood contamination, the absence of sequencing to validate species-level identification, the risk of false positives, and the lack of long-term outcome data.

## 7. Recommendations

Future large-scale studies are recommended to utilize advanced molecular techniques (such as quantitative PCR) and confirmatory culture methods to validate these findings. Furthermore, given the potential of serum PCR as a less invasive diagnostic tool, its clinical utility should be further explored and validated for use in resource-limited settings. Since our study did not include neonates because of methodological limitations, neonatal studies are needed to address the diagnostic problems in this highest-risk category.

## Figures and Tables

**Table 1:** Primers specific to the target organisms used in this study.

Organism	Target Gene	Oligo Name	Sequence (5′ to 3′)	Amplicon Size (bp) *	References
*N. meningitidis*	*CtrA*	*CtrA*-F	GCTGCGGTAGGTGGTTCAA	111	[20]
		*CtrA*-R	TTGTCGCGGATTTGCAACTA		
*S. pneumoniae*	*LytA*	*LytA*-F	ACGCAATCTAGCAGATGAAGCA	75	[21]
		*LytA*-R	TCGTGCGTTTTAATTCCAGCT		
*H. influenzae*	*Hpd*	*Hpd*-F	GGTTAAATATGCCGATGGTGTTG	151	[22]
		*Hpd*-R	TGCATCTTTACGCACGGTGTA		
*E. coli*	*rRNA*	*Eco*-F*rRNA*	GGGAGTAAAGTTAATACCTTTGC	204	[21]
		Eco-Rr*RNA*	CTCAAGCTTGCCAGTATCAG		
*L. monocytogenes*	*Hly*	Lm-F*hly*	CAT GGCACCACCAGC ATCT	64	[21]
		Lm-R*hly*	ATCCGCGTGTTTCTTTTCGA		
*S. aureus*	*FemA*	Sau-F*femA*	TGCTGGTGGTACATCAAA	97	[21]
		Sau-R*femA*	ACGGTCAATGCCATGATTTAA		
*S. agalactiae*	*Cfb*	Sag-F*cfb*	ATGATGTATCTATCTGGAACTCTAGTG	260	[21]
		Sag-R*cfb*	CGCAATGAAGTCTTTAATTTTTC		
*K. pneumoniae*	*rcsA*	Kpn-F*rcsA*	GGATATCTGACCAGTCGG	176	[23]
		Kpn-R*rcsA*	GGGTTTTGCGTAATGATCTG		

*(bp)* *—*base pair*.

**Table 2:** Distribution of the study participants by sex and age at TASH and Y12HMC.

Variable	Category	Frequency (%)
**Sex**	Male	109 (54)
	Female	93 (46)
	Total	202 (100)
**Age**	<12 Months	134 (66.3)
	1–15 Years	44 (21.8)
	≥16 Years	24 (11.9)
	Total	202 (100)

**Table 3:** Clinical presentation of the study participants at TASH and Y12HMC.

Clinical Presentation	Status	
	Yes (%)	No (%)
Poor feeding	151 (74.8)	51 (25.2)
High fever (≥38.5 °C)	144 (71.3)	58 (28.7)
Loss of consciousness	134 (66.3)	68 (33.7)
Neck stiffness	58 (28.7)	144 (71.3)
Seizures	60 (29.7)	142 (70.3)
Bulging fontanelle of an infant	23 (11.4)	179 (88.6)
Petechiae (skin rash)	5 (2.5)	177 (97.5)

**Table 4:** CSF findings of WBC counts, Gram staining, culture, and history of antibiotic use.

Conventional Laboratory Analysis	Appearance	Frequency (%)
Cell in CSF (≥10 cell/mm^3^)	Yes	52 (25.7)
	No	150 (74.3)
	Total	202 (100)
Gram stain	Negative	191 (94.6)
	Positive	11 (5.4)
	Total	202 (100)
Culture result	Negative	193 (95.5)
	Positive	9 (4.5)
	Total	202 (100)
History of antibiotic usage before admission	Yes	140 (69.3)
	No	62 (30.7)
	Total	202 (100)
History of vaccination	Yes	59 (29.2)
	No	143 (70.8)
	Total	202 (100)

**Table 5:** Comparison of pathogens detected via CSF and serum PCR tests at TASH and Y12HMC.

	CSF	Serum		Total
		Negative	Positive	
*N. meningitidis*	Negative	194	1	195
	Positive	4	3	7
	Total	198	4	202
*S. pneumoniae*	Negative	172	22	194
	Positive	3	5	8
	Total	175	27	202
*H. influenzae*	Negative	192	5	197
	Positive	4	1	5
	Total	196	6	202
*S. agalactiae*	Negative	200	1	201
	Positive	1	0	1
	Total	201	1	202
*E. coli*	Negative	132	40	172
	Positive	13	17	30
	Total	145	57	202
*S. aureus*	Negative	197	4	201
	Positive	1	0	1
	Total	198	4	202
*L. monocytogenes*	Negative	201	1	202
	Positive	0	0	0
	Total	201	1	202
*K. pneumoniae*	Negative	196	3	199
	Positive	3	0	3
	Total	199	3	202

**Table 6:** *McNemar’s* test of comparison between CSF and serum PCR tests of study participants at TASH and Y12HMC.

	Positive Serum	Negative Serum	Total
Positive CSF	34	20	54
Negative CSF	59	89	148
Total	93	109	202

McNemar’s Test—Test statistic = 18.27848, *p*-value (1 tail) = 0.00001, *p*-value (2 tails) = 0.00002, and *odds ratio* = 0.33898. Cohen’s Kappa: κ = 0.188 (95% CI: 0.109–0.293).

**Table 7:** Number of patients who received antibiotic treatment before sample collection and PCR test results.

PCR-Result	Positive	Negative	Total
CSF-PCR	39	101	140
Serum-PCR	60	80	140
Total	99	41	140

## Data Availability

The raw data supporting the conclusions of this article will be made available by the authors on request.

## Abbreviations

The following abbreviations are used in this manuscript:

AHRI	Armauer Hansen Research Institute
BM	Bacterial meningitis
CLSI	Clinical & Laboratory Standards Institute
CSF	Cerebrospinal fluid
Hib	*Haemophilus influenzae* type b
LMIC	Low and middle-income countries
LP	Lumbar puncture
PCR	Polymerase chain reaction
SPSS	Statistical Package for the Social Sciences
TASH	Tikur Anbessa Specialized Hospital
Y12HMC	Yekatit 12 Hospital Medical College

## References

[1] Bijlsma M.W., Brouwer M.C., Kasanmoentalib E.S., Kloek A.T., Lucas M.J., Tanck M.W., van der Ende A., van de Beek D. (2016). Community-acquired bacterial meningitis in adults in the Netherlands, 2006–2014: A prospective cohort study. Lancet Infect. Dis..

[2] Welinder-Olsson C., Dotevall L., Hogevik H., Jungnelius R., Trollfors B., Wahl M., Larsson P. (2007). Comparison of broad-range bacterial PCR and culture of cerebrospinal fluid for diagnosis of community-acquired bacterial meningitis. Clin. Microbiol. Infect..

[3] Bronska E., Kalmusova J., Dzupova O., Maresova V., Kriz P., Benes J. (2006). Dynamics of PCR-based diagnosis in patients with invasive meningococcal disease. Clin. Microbiol. Infect..

[4] Amare A.T., Kebede Z.T., Welch H.D. (2018). Epidemiology of bacterial meningitis in children admitted to Gondar University Hospital in the post pneumococcal vaccine era. Pan Afr. Med. J..

[5] Mihret W., Lema T., Merid Y., Kassu A., Abebe W., Moges B., Tenna A., Woldegebriel F., Yidnekachew M., Mekonnen W. (2016). Surveillance of Bacterial Meningitis, Ethiopia, 2012–2013. Emerg. Infect. Dis..

[6] Mesele D.W., Kalayu A.A., Teshome S., Aman J., Alemu A., Ayele L., Gelaw A., Mihret A., Mulu A., Alemayehu D.H. (2025). Enhanced detection of bacterial etiologies, antibiotic resistance profile, and treatment outcomes of meningitis patients in Ethiopia: A prospective cross-sectional study. BMC Microbiol..

[7] Awulachew E., Diriba K., Awoke N. (2020). Bacterial Isolates from CSF Samples and Their Antimicrobial Resistance Patterns Among Children Under Five Suspected to Have Meningitis in Dilla University Referral Hospital. Infect. Drug Resist..

[8] Desmond N.A., Nyirenda D., Dube Q., Mallewa M., Molyneux E., Lalloo D.G., Heyderman R.S. (2013). Recognising and treatment seeking for acute bacterial meningitis in adults and children in resource-poor settings: A qualitative study. PLoS ONE.

[9] Scarborough M., Thwaites G.E. (2008). The diagnosis and management of acute bacterial meningitis in resource-poor settings. Lancet Neurol..

[10] Dubos F., Lamotte B., Bibi-Triki F., Moulin F., Raymond J., Gendrel D., Bréart G., Chalumeau M. (2006). Clinical decision rules to distinguish between bacterial and aseptic meningitis. Arch. Dis. Child..

[11] Neuman M.I., Tolford S., Harper M.B. (2008). Test characteristics and interpretation of cerebrospinal fluid gram stain in children. Pediatr. Infect. Dis. J..

[12] Obaro S. (2019). Updating the diagnosis of bacterial meningitis. Lancet Infect. Dis..

[13] Albuquerque R.C., Moreno A.C.R., Dos Santos S.R., Ragazzi S.L.B., Martinez M.B. (2019). Multiplex-PCR for diagnosis of bacterial meningitis. Braz. J. Microbiol..

[14] Diallo K., Feteh V.F., Ibe L., Antonio M., Caugant D.A., du Plessis M., Deghmane A.E., Feavers I.M., Fernandez K., Fox L.M. (2021). Molecular diagnostic assays for the detection of common bacterial meningitis pathogens: A narrative review. eBioMedicine.

[15] Newcombe J., Cartwright K., Palmer W.H., McFadden J. (1996). PCR of peripheral blood for diagnosis of meningococcal disease. J. Clin. Microbiol..

[16] Bårnes G.K., Gudina E.K., Berhane M., Abdissa A., Tesfaw G., Abebe G., Feruglio S.L., Caugant D.A., Jørgensen H.J. (2018). New molecular tools for meningitis diagnostics in Ethiopia—A necessary step towards improving antimicrobial prescription. BMC Infect. Dis..

[17] WHO (2018). Standard Operating Procedures for Surveillance of Meningitis Preparedness and Response to Epidemics in Africa.

[18] Institute EHaNR (2013). National Guideline on Meningococcal Meningitis Surveillance.

[19] Prevention WHOCfDCa (2011). Laboratory Methods for the Diagnosis of Meningitis Caused by Neisseria Meningitidis, Streptococcus Pneumoniae, and Haemophilus Influenzae.

[20] Corless C.E., Guiver M., Borrow R., Edwards-Jones V., Fox A.J., Kaczmarski E.B. (2001). Simultaneous detection of Neisseria meningitidis, Haemophilus influenzae, and Streptococcus pneumoniae in suspected cases of meningitis and septicemia using real-time PCR. J. Clin. Microbiol..

[21] Wang Y., Guo G., Wang H., Yang X., Shao F., Yang C., Gao W., Shao Z., Zhang J., Luo J. (2014). Comparative study of bacteriological culture and real-time fluorescence quantitative PCR (RT-PCR) and multiplex PCR-based reverse line blot (mPCR/RLB) hybridization assay in the diagnosis of bacterial neonatal meningitis. BMC Pediatr..

[22] Wang X., Mair R., Hatcher C., Theodore M.J., Edmond K., Wu H.M., Harcourt B.H., Carvalho Mda G., Pimenta F., Nymadawa P. (2011). Detection of bacterial pathogens in Mongolia meningitis surveillance with a new real-time PCR assay to detect Haemophilus influenzae. Int. J. Med. Microbiol..

[23] Dong D., Liu W., Li H., Wang Y., Li X., Zou D., Yang Z., Huang S., Zhou D., Huang L. (2015). Survey and rapid detection of Klebsiella pneumoniae in clinical samples targeting the rcsA gene in Beijing, China. Front. Microbiol..

[24] McHugh M.L. (2012). Interrater reliability: The kappa statistic. Biochem. Med..

[25] MedCalc Software Ltd. (2025). Comparison of Proportions. MedCalc Online Statistical Calculator. https://www.medcalc.org/en/calc/comparison_of_proportions.php.

[26] Ahmed M.A., Askar G.A., Farghaly H.S., Ahmed A.O., Kamal D.T., Ahmed S.S., Mohamad I.L. (2022). Accuracy of cerebrospinal fluid C-reactive protein and multiplex polymerase chain reaction and serum procalcitonin in diagnosis of bacterial and viral meningitis in children. Acta Neurol. Taiwanica.

[27] Sharma N., Gautam H., Tyagi S., Raza S., Mohapatra S., Sood S., Dhawan B., Kapil A., Das B.K. (2022). Clinical use of multiplex-PCR for the diagnosis of acute bacterial meningitis. J. Fam. Med. Prim. Care.

[28] Raza S.D.B., Chaudhry R., Goyal V., Lodha R., Sood S., Gautam H., Kapil A. (2022). Efficiency of Real-Time PCR in the Diagnosis of Community-Acquired Bacterial Meningitis in Children. J. Microbiol. Infect. Dis..

[29] Geteneh A., Kassa T., Alemu Y., Alemu D., Kiros M., Andualem H., Abebe W., Alemayehu T., Alemayehu D.H., Mihret A. (2020). Enhanced identification of Group B streptococcus in infants with suspected meningitis in Ethiopia. PLoS ONE.

[30] Airede K.I., Adeyemi O., Ibrahim T. (2008). Neonatal bacterial meningitis and dexamethasone adjunctive usage in Nigeria. Niger. J. Clin. Pract..

[31] Laving A.M., Musoke R.N., Wasunna A.O., Revathi G. (2003). Neonatal bacterial meningitis at the newborn unit of Kenyatta National Hospital. East Afr. Med. J..

[32] Biset S., Benti A., Molla L., Yimer S., Cherkos T., Eyayu Y., Ebabu A., Kasew D., Ambachew A. (2021). Etiology of Neonatal Bacterial Meningitis and Their Antibiotic Susceptibility Pattern at the University of Gondar Comprehensive Specialized Hospital, Ethiopia: A Seven-Year Retrospective Study. Infect. Drug Resist..

[33] Freedman S.B., Marrocco A., Pirie J., Dick P.T. (2001). Predictors of bacterial meningitis in the era after Haemophilus influenzae. Arch. Pediatr. Adolesc. Med..

[34] Feldman W.E. (1976). Concentrations of bacteria in cerebrospinal fluid of patients with bacterial meningitis. J. Pediatr..

[35] Tunkel A.R., Hartman B.J., Kaplan S.L., Kaufman B.A., Roos K.L., Scheld W.M., Whitley R.J. (2004). Practice guidelines for the management of bacterial meningitis. Clin. Infect. Dis..

[36] Tegene B., Gebreselassie S., Fikrie N. (2015). Bacterial Meningitis: A five-year retrospective study among patients who had attended at University of Gondar Teaching Hospital, Northwest Ethiopia. Biomed. Res. Ther..

[37] Mulu A., Kassu A., Tassema B. (2005). Bacterial isolates from cerebrospinal fluids and their antibiotic susceptibility patterns in Gondar University Teaching Hospital, Northwest Ethiopia. Ethiop. J. Health Dev..

[38] Daka D., Loha E., Giday A. (2011). Streptococcus pneumonia and antimicrobial resistance, Hawassa referral hospital, South Ethiopia. J. Med. Lab. Diagn..

[39] Assegu Fenta D., Lemma K., Tadele H., Tadesse B.T., Derese B. (2020). Antimicrobial sensitivity profile and bacterial isolates among suspected pyogenic meningitis patients attending at Hawassa University Hospital: Cross-sectional study. BMC Microbiol..

[40] Sonavane A.E., Baradkar V., Mathur M. (2008). Pattern and antibiotic susceptibility of bacteria isolated in clinically suspected cases of meningitis in children. J. Pediatr. Neurosci..

[41] Chugh Y., Kapoor A.K., Bhargava A. (2011). Antimicrobial sensitivity pattern of gram positive CSF isolates in children with septic meningitis in a Tertiary Care Hospital. Internet J. Med. Update.

[42] Britz E., Perovic O., von Mollendorf C., von Gottberg A., Iyaloo S., Quan V., Chetty V., Sriruttan C., Ismail N.A., Nanoo A. (2016). The Epidemiology of Meningitis among Adults in a South African Province with a High HIV Prevalence, 2009–2012. PLoS ONE.

[43] Gituro C.N., Nyerere A., Ngayo M.O., Maina E., Githuku J., Boru W. (2017). Etiology of bacterial meningitis: A cross-sectional study among patients admitted in a semi-urban hospital in Nairobi, Kenya. Pan Afr. Med. J..

[44] van Veen K.E., Brouwer M.C., van der Ende A., van de Beek D. (2016). Bacterial meningitis in diabetes patients: A population-based prospective study. Sci. Rep..

[45] Khan F.Y., Abu-Khattab M., Almaslamani E.A., Hassan A.A., Mohamed S.F., Elbuzdi A.A., Elmaki N.Y., Anand D., Sanjay D. (2017). Acute Bacterial Meningitis in Qatar: A Hospital-Based Study from 2009 to 2013. BioMed Res. Int..

